# Functional Frequency Discrimination From Cortical Somatosensory Stimulation in Humans

**DOI:** 10.3389/fnins.2019.00832

**Published:** 2019-08-07

**Authors:** Daniel R. Kramer, Krista Lamorie-Foote, Michael Barbaro, Morgan Lee, Terrance Peng, Angad Gogia, Charles Y. Liu, Spencer S. Kellis, Brian Lee

**Affiliations:** ^1^Department of Neurosurgery, University of Southern California, Los Angeles, CA, United States; ^2^Neurorestoration Center, University of Southern California, Los Angeles, CA, United States; ^3^Keck School of Medicine, University of Southern California, Los Angeles, CA, United States; ^4^Department of Biology and Biological Engineering, California Institute of Technology, Pasadena, CA, United States; ^5^Tianqiao and Chrissy Chen Brain-Machine Interface Center, California Institute of Technology, Pasadena, CA, United States

**Keywords:** somatosensation, cortical stimulation, brain computer interface, brain machine interface, sensory feedback control, electrocorticography, frequency

## Abstract

Recently, efforts to produce artificial sensation through cortical stimulation of primary somatosensory cortex (PSC) in humans have proven safe and reliable. Changes in stimulation parameters like frequency and amplitude have been shown to elicit different percepts, but without clearly defined psychometric profiles. This study investigates the functionally useful limits of frequency changes on the percepts felt by three epilepsy patients with subdural electrocorticography (ECoG) grids. Subjects performing a hidden target task were stimulated with parameters of constant amplitude, pulse-width, and pulse-duration, and a randomly selected set of two frequencies (20, 30, 40, 50, 60, and 100 Hz). They were asked to decide which target had the “higher” frequency. Objectively, an increase in frequency differences was associated with an increase in perceived intensity. Reliable detection of stimulation occurred at and above 40 Hz with a lower limit of detection around 20 Hz and a just-noticeable difference estimated at less than 10 Hz. These findings suggest that frequency can be used as a reliable, adjustable parameter and may be useful in establishing settings and thresholds of functionality in future BCI systems.

## Introduction

For the millions of patients with somatosensory deficits from stroke, paralysis, or limb-loss, restoration of function has vast implications for health, and independence recovery. Somatosensory brain-computer interface (BCI) presents a means to restore function in such individuals, where somatosensory input can potentially improve motor BCI (Andersen et al., 2004, 2010; Suminski et al., 2010; Fifer et al., 2012; Chestek et al., 2013; Lee et al., 2013; Aflalo et al., 2015; Bundy et al., 2016; Flesher et al., 2016; Hollins and Risner, 2016; Armenta Salas et al., 2018), or restore basic functions like bladder control. Although stimulation of peripheral nerves can potentially reproduce somatosensation (Raspopovic et al., 2014; Tan et al., 2014), to fully restore function in stroke patients or paralyzed individuals, cortical stimulation would be required. However, somatosensory BCI is at an early stage with limited work establishing the basic utility (Flesher et al., 2016; Armenta Salas et al., 2018; Lee et al., 2018), functionality (Baumgartner et al., 1991; Tan et al., 2014; Collins et al., 2017), and modalities (Raspopovic et al., 2014; Tan et al., 2014; Vidal et al., 2016; Collins et al., 2017; Armenta Salas et al., 2018; Lee et al., 2018).

Trained non-human primate (NHP) studies have helped to establish foundational parameters and feasibility of discriminating between sensations arising from intracortical micro-stimulation (ICMS) with microelectrodes in the primary somatosensory cortex (PSC). These techniques have produced behavioral responses comparable to those produced by real tactile stimuli (Romo et al., 1998, 2000; O’Doherty et al., 2012; Klaes et al., 2014). Through alterations in amplitude and location of an ICMS, NHPs have demonstrated an ability to differentiate pressure, location, and timing on par with that of tactile stimulation. By varying frequency in ICMS (10–30 Hz, with a minimum absolute difference in comparative frequencies of 2 Hz), NHPs showed similar accuracy to natural sensation produced by mechanical stimulation (80% vs. 89% accuracy) (Romo et al., 1998, 2000). O’Doherty et al. (2012) demonstrated that NHPs could differentiate between periodic and aperiodic pulse-trains in an active exploration task, suggesting that temporally patterned stimulation can lead to noticeable, unique sensations. Stimulation as low as 6 Hz produced perceptible sensations based on behavioral responses (Romo et al., 2000). They also identified that quickly adapting neurons were important in frequency discrimination, and discrimination between sensations was based on frequency alone. When slowly adapting neurons were stimulated, monkeys’ performances decreased and were not comparable to mechanical stimulation (Romo et al., 2000).

While NHP studies have demonstrated that artificial sensation through cortical stimulation is achievable (O’Doherty et al., 2011; Tabot et al., 2013, 2015; Kim et al., 2015a,b; Overstreet et al., 2016), clinical studies in humans are needed to fully understand the subjective quality of sensations evoked by modulating these parameters and verify the generalizability. Direct electrical stimulation of PSC, with ICMS and surface electrodes, have yielded reliable and safe results (Flesher et al., 2016; Armenta Salas et al., 2018; Lee et al., 2018) and established basic stimulation parameters (Lee et al., 2018). Changes in amplitude and frequency are primarily perceived as increased intensity, with occasional enlargement of dermatomal areas involved, and rarely a change in perceptual quality (Armenta Salas et al., 2018; Lee et al., 2018). Amplitude increase was most consistent in producing increased intensity (Lee et al., 2018). A study utilizing ECoG electrodes at three frequencies (50, 75, and 100 Hz) exemplified that sensation arising from stimulation at different frequencies can be differentiated in humans when there is at least a 25 Hz difference between frequencies and frequencies are greater than 50 Hz (Johnson et al., 2013). In addition, another high density ECoG study noted that perceiving somatosensation was inconsistent below 20 Hz for absolute perceptual threshold from a single stimuli (Lee et al., 2018). Although lower vibrotactile frequencies are better sensed in the skin (Vardar and Guclu, 2017), and have a wider range of responses (Griffin, 2012), earlier work by this group established a lower threshold in cortical stimulation of 20 Hz in which greater than 50% sensed the stimulation, and subject-described percepts reflected increased intensity and speed from increased cortical stimulation frequencies (Lee et al., 2018). Since the results of cortical stimulation on individual neurons is not well understood, the difference in frequency ranges and percepts likely reflects a difference in how cortical stimulation is interpreted compared to skin-sensed stimulation.

Going forward with BCI, establishing the psychometric thresholds for parameters like frequency will set the groundwork for what “degrees of freedom” are possible in subjects. Here we attempt to establish the perceptual limits of one specific parameter, frequency, during direct cortical stimulation of PSC using ECoG grids in humans with intact somatosensory pathways. We aim to estimate lower limits, and reliable degrees of freedom for frequency changes in stimulation parameters, for the purpose of using ECoG as a delivery method in somatosensory BCI in the future. We also aim to explore the usable limits for detecting the absolute difference between two frequencies.

## Materials and Methods

### Subjects and Implantation

Three patients with intractable epilepsy, of normal intelligence on neuropsychiatric testing, normal somatosensation, and undergoing implantation of subdural ECoG with coverage over PSC, were enrolled in this study (see Table 1 for demographic details). These patients, as part of their care for epilepsy, required ECoG for seizure localization, and were to receive a craniotomy with access to the PSC hand region. S12 had a cavernous malformation in the parietal lobe. Based on the mapping of PSC, imaging, and results from invasive monitoring, the cavernoma was separate from the PSC hand area, and the seizure focus was not near PSC. S18 was found to have a seizure focus in the interhemispheric portion of the parietal lobe, also distinct from PSC. S30 was found to have seizure foci in the frontal and temporal lobes, also distinct from PSC. This study was approved by the USC Institutional Review Board and all subjects provided written consent. Surgical technique was standard, and has been described elsewhere (Lee et al., 2018), but briefly, a craniotomy was to be performed with access to the frontal, temporal, and parietal regions. Prior to surgery, the motor cortex hand area was identified based on anatomic landmarks, and the hand representation in PSC was marked, using neuronavigation software. During surgery, grids were placed to center over the PSC hand area. This area was not under direct visualization. Grids were high-density, “mini”-ECoG grids (mECoG) with 2 mm contacts, with 1.2 mm exposed surface of platinum-iridium electrodes between silastic sheeting, spaced 3 mm apart from center-to-center (FG64C-MP03, Ad-Tech Medical Instrument Corporation, Wisconsin, WI, United States), except in S12 where a standard spaced ECoG grid (sECoG) was used with 4 mm contacts, with 2.4 mm exposed surface, spaced 10 mm apart from center-to-center (AU4 × 5P2, Integra Life Sciences Corporation, New Jersey, United States). Following implantation, the grids were secured to the dura, tunneled through the scalp, and secured to the scalp with suture. The dura was closed, bone replaced, and scalp closed; the patient was transported to the epilepsy monitoring unit in the intensive care unit (ICU).

**TABLE 1:** Patient Demographics.

**Subject**	**Seizure foci**	**Radiographic abnormalities**	**Epilepsy duration (years)**	**Age (years)**	**Sex**	**Dominant hand**	**Dermatome chosen for stimulation**
S12	Right parietal	Prior surgery for right parietal cavernous malformation	3	25	M	R	Digit 5 medial surface
S18	Posterior interhemispheric strip, lateral parietal	N/A	11	32	F	R	Medial palm
S30	Left interhemispheric frontal cortex in area of encephalomalacia	Left frontal/temporal encephalomalacia	11	24	F	R	Medial palm and wrist

*Subjects were epilepsy patients who underwent subdural electrode placement for the purpose of seizure localization. An electrocorticography grid was placed over primary somatosensory cortex. Dermatomes for stimulation testing were chosen after mapping by an epileptologist, where the bipolar electrodes had stable dermatomal percepts upon multiple repeat stimulations.*

### Experimental Set Up

**FIGURE 1:**
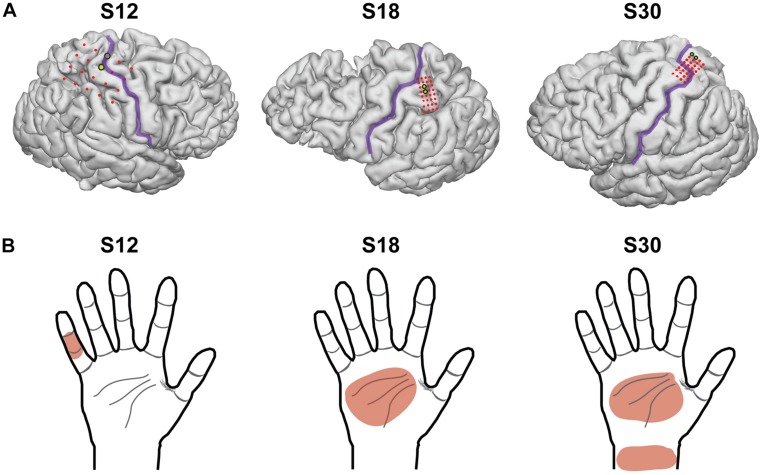
Electrocorticography grid placement. **(A)** 3-dimensional images of each subject’s brain from a magnetic resonance image, with the location of the electrodes superimposed. The central sulcus is outlined in purple and electrodes of the grid are shown in red. The electrodes chosen for stimulation in this experimental model are highlighted in yellow. S12 had electrodes with 1 cm of spacing, and S18 and S30 had electrodes with 3 mm of spacing (center-to-center). **(B)** The dermatomal distribution of the percepts used for testing after grid mapping for each subject.

Location of the implanted grid was confirmed by imaging with a computed tomography scan fused to a preoperative magnetic resonance imaging scan (Figure 1). Superimposition of grid placement was made using Freesurfer and Statistical Parametric Mapping software SPM12 with the *img_pipe* package described in Hamilton et al. (2017). Functional location of electrodes was confirmed by mapping with cortical stimulation while subjects were in the ICU. Electrode pairs were stimulated with a range of amplitudes, at the discretion of the epileptologist, between 0.5 and 12 mA, with a frequency of 50 Hz, pulse-width of 250 μs, and duration of 1 s. Areas with pure sensory responses (self-reported by the subject), underwent steady increases in amplitude until motor responses were noted. Following mapping, electrode pairs with sensory only responses at 4 mA were retested 25 times to confirm that repeat stimulation did not (1) alter the percept by the subject, (2) alter the location or region of perception, (3) result in motor activity, (4) cause seizures or seizure-like activity, or (5) cause discomfort. All subjects had at least one electrode pair that met these criteria. If more than one electrode pair met these criteria, there was a preference for the ventral side of the hand and for digits on the lateral side of the hand over the medial (see Table 1). This electrode pair was then chosen for repeat stimulation with our paradigm.

**FIGURE 2:**
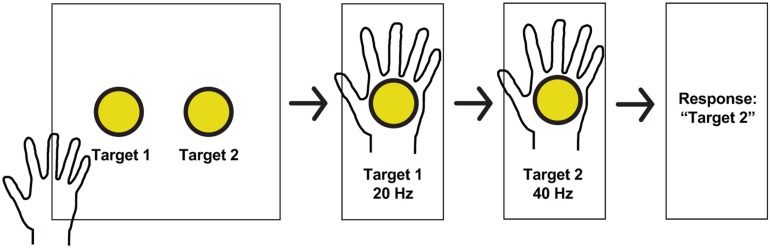
Experimental paradigm and hand receptive fields. Schematic of a typical session. Subjects received stimulation at one of two pseudorandomly determined frequencies when searching for targets in a 2-dimensional space, and reported which target had the higher frequency, whether or not they guessed, and whether or not both stimulations were perceived.

Subjects explored a 2-dimensional space consisting of a sheet with two circles on it, which the subject placed at a comfortable distance for exploring with the hand contralateral to the implanted grid. The two circles corresponded to two different stimulation frequencies. As the subject moved one hand over the two circles, an epileptologist stimulated PSC with the associated frequency using an FDA-approved, clinically available stimulator (Natus Neurology Incorporated, Warwick, United States). The current, pulse-width, and pulse-duration were held stable (4 mA, 250 μs, and 1 s, respectively). In a two-alternative forced-choice task design, subjects were instructed to report which circle corresponded to the higher frequency, whether they were “guessing” (meaning they were not sure which one was the higher frequency), and whether they felt both stimulations (see Figure 2 for experimental setup). Frequencies included 20, 30, 40, 50, 60, and 100 Hz and were chosen pseudorandomly. The stimulator did not have parameters between 60 and 100 Hz. Because subjects moved at their own speed, the time between stimuli ranged from 1 to 6 s. Statistical analysis was performed using Matlab software (The Mathworks, Natick, MA, United States). Thresholds and differences in detection were compared using Fisher’s exact test.

## Results

Electrodes used for stimulation were determined after cortical mapping and somatosensory percepts were reported by the subject and remained stable after repeat stimulations (>50 stimulations). Stimulation parameters included pulse-width of 250 μs, duration of 1 s, and a square-wave, and were chosen based on prior literature (Lee et al., 2018) and to minimize the interplay of the other parameters with frequency (i.e., low, but reliably detected on 25 repeat stimulations at 50 Hz). Amplitude was chosen to be the lowest value that elicited reliable somatosensory percepts on the 50 prior stimulations with a 50 Hz frequency, and that did not elicit motor activity (2 mA for S12, 3 mA for S18, and 5 mA for S30). Selected dermatomes were the medial surface of digit 5 for S12, medial surface of the palm for S18, and medial palm and wrist for S30 (Table 1). Twenty-five trials were completed for S12 and 50 trials for S18 and S30. For S12, 50 trials were planned, however, the patient chose to stop half-way through testing due to fatigue. No adverse events occurred. With increased frequency, patients described the sensation as “more intense,” “faster,” and “faster buzzing.”

### Overall Accuracy

**FIGURE 3:**
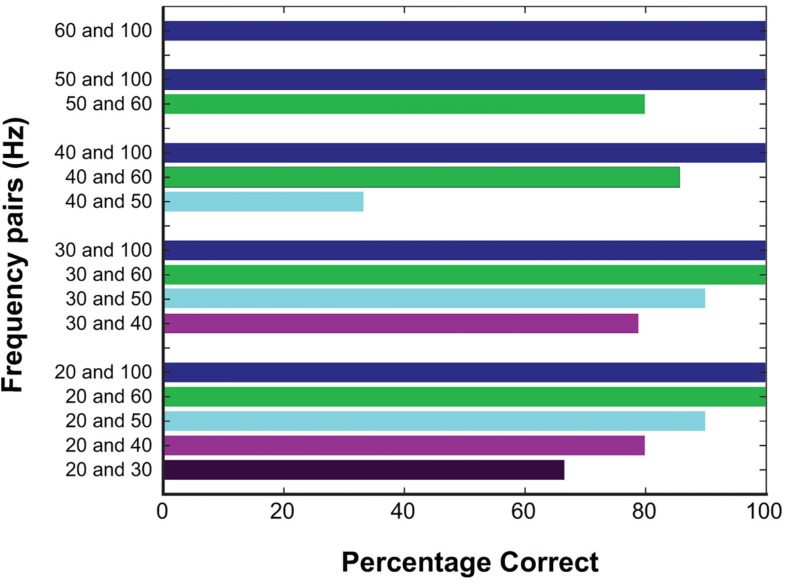
Responses at different frequencies tested. Percentage correct for all trials based on the frequencies being compared, color coded based on the higher frequency. Correct responses were high with larger frequency differences.

Altogether, participants identified the higher frequency with 89.76% accuracy. To explore whether the first stimulation might alter the perception of the second stimulation, trials were grouped based on which condition occurred first. Correct responses were statistically equivalent between these groups (91.9% when the higher frequency occurred first vs. 86.8% when it occurred second; *p* = 0.39, Fisher’s exact test). The accuracy of trials broken down into the individual categories is included in Figure 3.

**FIGURE 4:**
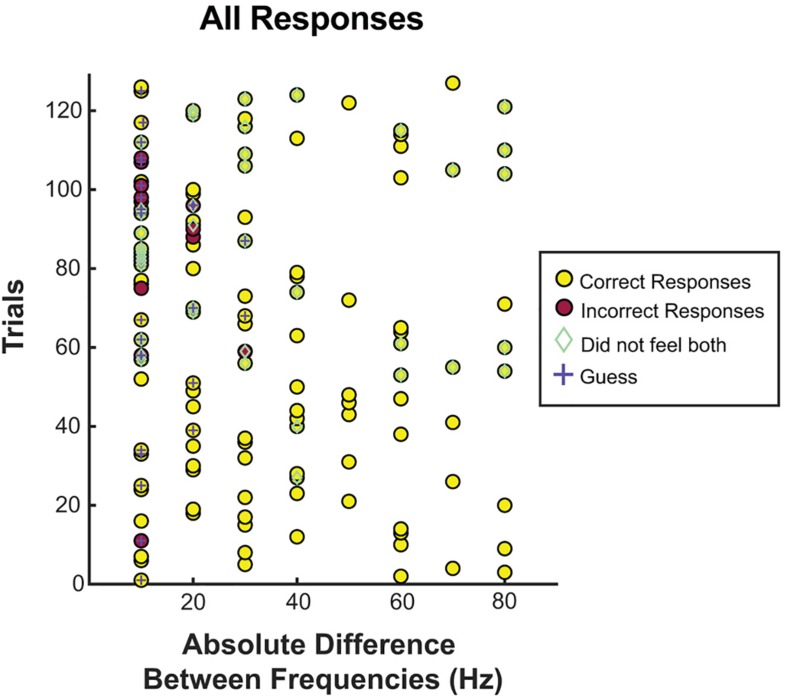
Correct responses organized by absolute difference between two frequencies. All trials separated by the absolute difference in frequency. Correct and incorrect responses, guessing, and whether or not the patient felt both stimuli were recorded for each frequency difference. Most incorrect trials, guessing, and inability to feel both frequencies occurred at or below a difference of 20 Hz.

**FIGURE 5:**
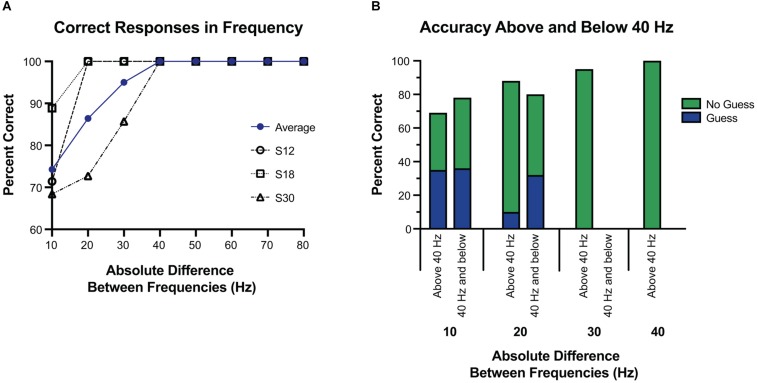
Accuracy at tested frequency differences. **(A)** Overall accuracy for each subject, at each frequency difference. The average of their results is in blue. Above a 30 Hz difference, accuracy was 100%, and well above chance at or below 30 Hz. **(B)** Response accuracy and guessing rate when the tested frequencies were both 40 Hz and below vs. when one or more of the tested frequencies was above 40 Hz. Here the blue and green represent which portions of correct responses were self-reported as a guess, or not a guess, respectively. Overall, subjects were equally accurate regardless of whether the frequencies were above or below 40 Hz, and were more likely to guess if the difference was 10 Hz.

Next, we focused on the absolute difference between two frequencies (see Figures 4, 5). When the frequency difference was equal to 10 Hz, the mean accuracy was 74.29% (S12 71.4%; S18 88.9%; S30 68.4%, see Figure 5) compared to a difference larger than 10 Hz, where accuracy increased to 95.65% (*p* < 0.001, individually: S12 100%; S18 100%; S30 87.1%). For S12 and S18, incorrect responses only occurred at a difference of 10 Hz, when both frequencies were equal to or less than 40 Hz.

Using 40 Hz as a cutoff of lower frequencies and higher frequencies, we examined whether small absolute differences were easier to differentiate at lower frequencies or higher frequencies. Separating the trials into those with both frequencies 40 Hz or less vs. those with either frequency greater than 40 Hz, accuracy was 77.8% vs. 93.0% (*p* < 0.05). However, this difference was largely explained by the larger absolute differences of frequencies above 40 Hz. Comparing the absolute difference of 20 Hz or less (since the frequencies at or below 40 Hz had a max absolute difference of 20 Hz) the accuracy was 77.8% vs. 80.0% (*p* = 1.0) for below 40 Hz and above 40 Hz, respectively. When isolating the trials in which the frequency difference was 10 Hz, and then separating them into those with both frequencies 40 Hz or less vs. those with one or both greater than 40 Hz, accuracy was 77.3% vs. 69.2% (*p* = 0.69), respectively. At a frequency difference of 20 Hz, accuracy was 80% when both frequencies were 40 Hz or less compared to 88.2% when at least one was greater than 40 Hz (*p* = 0.66) (Figure 5). Frequency differences of 20 Hz had an accuracy of 86.4%, and differences of 30 Hz had an accuracy of 95.0%. All other differences had an accuracy of 100% (see Figure 5).

### Patient Reported “Guessing”

Subjects reported when they were “guessing,” defined as when they could not tell which stimulus was higher. Of all trials, guessing occurred in 18.1% of trials. When subjects reported guessing, accuracy was 69.6% vs. 94.2% when they did not report guessing (*p* < 0.01). Limited to trials in which the difference was 20 Hz or less, guessing occurred 36.8% of the time, vs. 2.9% of the time when the difference was greater than 20 Hz (*p* < 0.001). Accuracy for only trials in which the difference was 20 Hz or less was 66.7% when guessing vs. 86.1% when not guessing (*p* = 0.10).

Further, to examine if guessing was more frequent when the frequencies were both low vs. when they were high, we split the 20 Hz differences into those both at or below 40 Hz vs. the rest (see Figure 4). For a difference of 20 Hz or less, when both frequencies were at or below 40 Hz, guessing occurred 48.1% of the time vs. 26.7% when one or more frequencies was above 40 Hz (*p* = 0.10), and correct responses were given 77.8% of the time at or below 40 Hz vs. 80.0% of the time above 40 Hz (*p* = 1.0).

When the difference was equal to 10 Hz, guessing occurred 48.6% of the time, vs. 6.5% of the time when the frequency difference was greater than 10 Hz (*p* < 0.001). Among trials with a difference of 10 Hz, accuracy was 64.7% when guessing vs. 83.3% when not guessing (*p* = 0.26). Again, we examined if having both frequencies lower than 40 Hz affected the rate of accuracy or guessing among those that were 10 Hz apart. No difference was seen among subjects’ rate of guessing when both frequencies were at or below 40 Hz vs. one or more above 40 Hz (46.1% vs. 50.0%, *p* = 1.0), or subjects’ accuracy when guessing (77.2% vs. 69.2%, *p* = 0.70).

### Thresholds of Detection

To understand the lower limits of detection thresholds, subjects were asked to report on whether they felt both stimulations, of which 69.3% of trials were reported as perceived for both stimulations (see Figure 4). Of the 30.7% in which one or more stimulations were not perceived, 82.1% had one or both frequencies at or below 30 Hz, and 92.3% had one or both frequencies at or below 40 Hz. Correct responses were not significantly different when subjects felt both stimulations (89.8% when both were felt vs. 89.7% when one or both were not felt). No difference was found in whether both stimulations were felt when split for both stimulation frequencies being below 50 Hz vs. both being above 50 Hz (60.0% vs. 75.3%, *p* = 0.079) or both being below 40 Hz vs. both being above 40 Hz (63.0% vs. 71.0%, *p* = 0.483).

## Discussion

Here we build upon prior work using ECoG electrodes to establish the thresholds of functionality for somatosensory BCI by adjusting frequency and discerning discrimination thresholds. The ability to detect a difference in frequency from cortical stimulation using an ECoG grid adds an adjustable parameter to the prospect of somatosensory BCI. To this end, we sought to explore the basic psychometric parameters of frequency discrimination in epilepsy patients with already implanted ECoG grids over PSC. Subjectively, when frequency increased, patients perceived a more intense stimulus, similar to other stimulation studies in humans (Johnson et al., 2013; Hiremath et al., 2017; Lee et al., 2018). Even when subjects reported guessing on a forced choice between stimulations of two different frequencies, accuracy was 69.6%. Finally, we show detection of absolute differences between stimulation frequencies above chance at 10 Hz, with reliable differentiation of frequencies at or above 20 Hz and near perfect detection at or above 40 Hz.

Prior ECoG work with cortical stimulation tested frequencies of 50, 75, and 100 Hz and noted that patients were able to identify and differentiate between sensations elicited by these frequencies (Johnson et al., 2013). Our own work showed that intensity was altered with an increase in frequency, but detecting differences was not explicitly tested (Lee et al., 2018). These studies investigating the frequency component of stimulation have mostly studied the feasibility and subjective quality of modulating frequency to produce different sensations, as well as the threshold required for somatosensory percepts (Johnson et al., 2013; Hiremath et al., 2017; Lee et al., 2018). Prior studies in microelectrode stimulation have focused on amplitude, demonstrating alterations in somatosensory category, with the higher amplitudes leading to more proprioceptive sensations, and lower amplitudes leading to cutaneous sensations (Armenta Salas et al., 2018). Further intracortical stimulation with microelectrodes in humans suggested that the “just-noticeable difference” (JND) of amplitude is around 15.4 ± 3.9 μA with a detection threshold of 39.4 μA (Flesher et al., 2016).

To establish similar baseline parameters in frequency alterations, we built off prior work. With preliminary testing we noted that frequencies below 20 Hz were generally undetectable (Lee et al., 2018), and thus explored 10 Hz differences from 20 to 100 Hz (except 70–90 Hz due to technical constraints). Above a 30 Hz difference, accuracy was 100%, with a high degree of accuracy at all tested differences below 30 Hz. Frequency differences of 20 Hz led to an accuracy of 86.4%, and differences of 10 Hz were still well above chance at 74.3%. S12 and S18 had only two incorrect responses each, both at a 10 Hz difference, with both frequencies at or below 40 Hz. S30 was more varied, but had most incorrect responses at a difference of 10 Hz (60%), and all were when both frequencies were less than 60 Hz.

Although it was more difficult to detect a difference of 10 Hz compared to 20 or 30 Hz, accuracy at a 10 Hz difference was well above chance, and thus we estimate a JND below 10 Hz for cortical stimulation through ECoG electrodes. One exception to this, in Figure 3, 40 Hz vs. 50 Hz has a 33.3% accuracy, and may reflect a limited number of trials at that frequency pair, or reflect a true difference. The edges of detection for Meissener corpuscles (flutter sensations at frequencies 10–60 Hz) and Pacinian afferents (vibrational sensations 60–400 Hz) are right around 40 and 50 Hz (Mountcastle et al., 1969), and therefore this may reflect a difficulty in this frequency range for both cortical and peripheral stimulation. Further testing may be warranted.

Weber’s law would indicate that the JND would increase proportionally to the increase in the base frequency. Indeed, Weber’s law is noted in studies comparing tactile somatosensation (Francisco et al., 2017), however it is not clear if this follows in cortical stimulation. Limited by the testing capabilities of our stimulator, we were unable to explore smaller differences, and thus unable to find a true JND, or the adherence of PSC stimulation to Weber’s law. In a NHP microstimulation study, the JND was 3.73 Hz with cortical stimulation (Romo et al., 2000), which may be closer to the real JND or may represent a difference in species, electrode type, or both. Compared to vibrational frequencies of mechanical stimuli, the JND was similar between the two (2.88 Hz for mechanical stimuli). JND could be different with ECoG electrode stimulation vs. microelectrode stimulation given changes in the spread of electricity (bipolar vs. unipolar), size of the electrode (mm vs. μm), or other differences (e.g., mA vs. μA). The JND estimated in this study (<10 Hz) is not far from the JNDs reported for microelectrode stimulation and mechanical stimuli, and although it may abide by Weber’s law, all that we can conclude here is that between 50 and 60 Hz (the largest base frequency with the smallest absolute difference), accuracy was well above chance at 80%. Since microelectrode, macroelectrode, and mechanical stimulation appear to have similar JNDs, it may be that the encoding of vibrational frequencies is tied to the frequency of the electrical activity in PSC whether from peripheral nerves or direct stimulation. Indeed, lower vibrational frequencies of mechanical stimuli (<100 Hz) have been seen to stimulate PSC neuron spike rates (Mountcastle et al., 1969; Salinas et al., 2000; Hernandez et al., 2005; Luna et al., 2005). Further testing with stimulators with more finely tuned adjustments will be necessary in the future, and may need to rely on concepts that compare tactile and cortical stimulation thresholds like “psychometric equivalence functions,” already shown to be able to estimate these thresholds in amplitude and frequency stimulations in rodents (Devecioglu and Guclu, 2005).

Subject-reported guessing was also evaluated, showing a low rate of guessing across all trials at 18.1%. Most guessing occurred at the lower range of differences, 10 and 20 Hz, however, accuracy was not significantly altered, and correct responses were above 50%. This result indicates that despite self-reports, subjects were still able to differentiate between frequencies. Similarly, subjects reported they could not feel both stimulations approximately 1/3 of the time. Again, this outcome did not diminish correct responses, which were similar when comparing those in which they reported feeling both to those in which they did not (see Figure 3). Ninety-two percent of the time that subjects did not sense the stimulation, one frequency was at or below 40 Hz. Taken together, accuracy decreased and guessing increased when both frequencies were less than 30 Hz, and inconsistencies were seen at 30 and 40 Hz. Given these results, 40 Hz is likely the threshold for consistent sensation, and would be a reasonable lower limit for a somatosensory BCI system. From these results, an ECoG BCI system would likely be able to manipulate frequencies at 20 Hz differences from 40 Hz and above, with a high degree of reliability. With training and more fine adjustments, differences of 10 Hz would likely be sufficient. We did see a difference between subjects, with one subject showing decreased accuracy above 30 Hz, suggesting intersubject variability. Additionally, the thresholds were based on fixed parameters of amplitude, pulse-width, and pulse-duration, all factors which independently (Lee et al., 2018), and jointly influence thresholds (Devecioglu and Guclu, 2005). Likely an increased amplitude or pulse-duration would alter the thresholds of frequency discrimination, as well as the perception of the stimulus [as evidenced by changes in the strength of the stimulus noted by Lee et al. (2018)]. A great deal more testing in future studies will be required to elucidate the psychophysical results of the combining parameter variations and a future BCI system will likely need a range of possible frequencies to adjust for individual variation.

This study has several limitations. First, the stimulator used, an FDA-approved clinical stimulator, can only test differences as small as 10 Hz. Therefore, while the “just-noticeable” difference most likely lies below 10 Hz, we were unable to stimulate smaller changes given that the stimulator settings are unalterable (see Figure 4). Similarly, the parameters are limited, without options between 60 and 100 Hz. Testing was carried out in the ICU, limiting time and control, and preventing more trials and further paradigms. Subjects all suffered from epilepsy, which may alter cortical networks in PSC. However, our subjects were still able to perceive sensation in a consistent dermatome upon stimulation and had no known pathology affecting PSC. The other stimulation parameters were not consistent between patients (but rather consistent within patients), introducing bias. Electrode size was different for S12 than S18 and S30, which may have introduced differences into the spread of electricity. Each session only took place in a single day and the results are therefore not generalizable to chronic stimulation. The stability of chronic stimulation for producing somatosensation is unknown, however, reports of chronic stimulation of motor cortex in NHPs shows stability of mapping (Craggs et al., 1976), the safety profile from chronically implanted, stimulating, and surface electrodes in responsive neural stimulators is quite robust (Heck et al., 1976; Bergey et al., 2015; Lee et al., 2015). ICMS use in PSC exhibits stability at 10–12 weeks, without any safety concerns. Since ICMS is invasive, it is likely that the safety profile would be similar or better, but the decay may be more severe (Callier et al., 2015; Chen et al., 2015).

Overall, this study uses ECoG grids to investigate the frequency component of cortical stimulation for use in BCI systems. We estimate a JND value near or below 10 Hz, and show that even when guessing, subjects could correctly identify the higher frequency better than chance. Furthermore, these findings suggest reliable discrimination above 40 Hz, with a difference between frequencies of 20 Hz or above. Future BCI systems utilizing cortical stimulation to produce artificial sensation can utilize frequency to produce a wider range of percepts, empowering users to make better use of artificial sensations.

## Ethics Statement

This study was carried out in accordance with the recommendations of the University of Southern California Health Sciences Campus Institutional Review Board with written informed consent from all subjects. All subjects gave written informed consent in accordance with the Declaration of Helsinki. The protocol was approved by the University of Southern California Health Sciences Campus Institutional Review Board.

## Author Contributions

DK, BL, SK, and CL conceived the original ideas and experiments. BL and DK carried out the experiments. All authors interpreted the data, edited the manuscript, provided critical feedback, and helped to shape the research and analysis. DK, KL-F, CL, BK, and SK led the writing of the manuscript.

## Conflict of Interest Statement

The authors declare that the research was conducted in the absence of any commercial or financial relationships that could be construed as a potential conflict of interest.
